# The impact of Cognitive Processing Therapy on stigma among survivors of sexual violence in eastern Democratic Republic of Congo: results from a cluster randomized controlled trial

**DOI:** 10.1186/s13031-018-0142-4

**Published:** 2018-02-12

**Authors:** S. M. Murray, J. Augustinavicius, D. Kaysen, D. Rao, L. K. Murray, K. Wachter, J. Annan, K. Falb, P. Bolton, J. K. Bass

**Affiliations:** 1 Johns Hopkins Bloomberg School of Public Health, Department of Mental Health, 624 N Broadway Street, Baltimore, MD 21205 USA; 2 Department of Psychiatry and Behavioral Sciences, University of Washington, Seattle, WA USA; 3 Department of Global Health, University of Washington, Seattle, WA USA; 4 University of Texas, School of Social Work, Austin, TX USA; 5 Research, Evaluation and Learning Unit, International Rescue Committee, New York, NY USA; 6 Harris School of Public Policy, University of Chicago, Chicago, USA; 7 Johns Hopkins Bloomberg School of Public Health, Department of International Health, Baltimore, MD USA

**Keywords:** Social stigma, Rape, Sexual violence, Sub-Saharan Africa, Psychotherapy, Cognitive therapy

## Abstract

**Background:**

Sexual violence is associated with a multitude of poor physical, emotional, and social outcomes. Despite reports of stigma by sexual violence survivors, limited evidence exists on effective strategies to reduce stigma, particularly in conflict-affected settings. We sought to assess the effect of group Cognitive Processing Therapy (CPT) on stigma and the extent to which stigma might moderate the effectiveness of CPT in treating mental health problems among survivors of sexual violence in the Democratic Republic of Congo.

**Methods:**

Data were drawn from 405 adult female survivors of sexual violence reporting mental distress and poor functioning in North and South Kivu. Women were recruited through organizations providing psychosocial support and then cluster randomized to group CPT or individual support. Women were assessed at baseline, the end of treatment, and again six months later. Assessors were masked to women’s treatment assignment. Linear mixed-effect regression models were used to estimate (1) the effect of CPT on feelings of perceived and internalized (felt) stigma, and (2) whether felt stigma and discrimination (enacted stigma) moderated the effects of CPT on combined depression and anxiety symptoms, posttraumatic stress, and functional impairment.

**Results:**

Participants receiving CPT experienced moderate reductions in felt stigma relative to those in individual support (Cohen’s D = 0.44, *p* = value = 0.02) following the end of treatment, though this difference was no longer significant six-months later (Cohen’s D = 0.45, *p* = value = 0.12). Neither felt nor enacted stigma significantly moderated the effect of CPT on mental health symptoms or functional impairment.

**Conclusions:**

Group cognitive-behavioral based therapies may be an effective stigma reduction tool for survivors of sexual violence. Experiences and perceptions of stigma did not hinder therapeutic effects of group psychotherapy on survivors’ mental health.

**Trial registration:**

ClinicalTrials.gov NCT01385163.

**Electronic supplementary material:**

The online version of this article (10.1186/s13031-018-0142-4) contains supplementary material, which is available to authorized users.

## Background

In conflict-affected areas of Eastern Democratic Republic of Congo (DRC), nearly 40% of women participating in a community survey reported an experience of sexual violence (SV) in the 16 years following the 1994 civil war [1]. SV is associated with serious physical health consequences, including gynecological injuries, unintended pregnancy, and sexually transmitted diseases [2]. SV also has extensive psychological effects; elevated rates of depression, post-traumatic stress disorder (PTSD), and suicidality are well documented among survivors in DRC and elsewhere [1, 3, 4].

Due to the stigma associated with rape and its effects, SV can also profoundly affect survivors’ relationships with their partners, family, and community [5, 6]. Link and Phelan define stigma as a social labeling and distancing process that occurs in the context of a power differential [7]. Stigma can be experienced as perceived negative attitudes (i.e. perceived stigma) or changes in the way individuals are treated in their communities (i.e. discrimination or enacted stigma). Enacted stigma has been reported by SV survivors in DRC as taking various forms [5, 6, 8]. For example, approximately a third of SV survivors reported abandonment by their husbands or communities due to their experience of SV [9]. Survivors may also internalize the stigma they experience, for instance, by developing feelings of shame [5, 11].

Further exploration of the stigma associated with SV in DRC demonstrates a complex interrelationship with mental health. SV survivors who are rejected by their families have been found to experience higher levels of posttraumatic stress and depression symptoms on average relative to those whom remain accepted within their families [8]. Findings among adolescents in DRC support that stigma may be a mechanism by which SV produces poor mental health outcomes [10]. Mental distress may also exacerbate the effects of stigma on survivors either by leading to additional stigma or by increasing social isolation [11]. For instance, posttraumatic stress symptoms among women living in conflict-affected areas of DRC were found to be associated with fewer visits from family and community members to women’s homes, but also fewer visits by affected women to others’ homes [12].

This interrelationship raises the possibility that interventions to improve mental health among SV survivors may also be effective in reducing stigma. Peer support and counseling for individuals who are the target of stigma have been identified as promising anti-stigma interventions [13, 14]. A recent systematic review of HIV-related stigma reduction interventions identified only seven evaluations of this type of program in high or low and middle-income settings; information and contact-based programs (where individuals have an opportunity to interact with someone living with HIV/AIDS) for the general community were far more common [15]. Overall, the majority of these studies found that stigma decreased over the course of the program, but not all used a validated measure of stigma and several studies included fewer than 100 individuals. Evaluation of the impact of mental health programs on stigma is also warranted to ensure that interventions for marginalized populations do not unintentionally cause harm.

Cognitive Processing Therapy (CPT) is an evidence-based psychotherapy specifically developed for survivors of rape that includes psychoeducation and cognitive restructuring [16]. We previously found that group-based CPT had large effect sizes (ranging from 1.1–1.8) on trauma, depression and anxiety, and functional impairment for survivors of sexual violence in DRC [17]. Group CPT was also observed to have small effects on emotional support seeking and membership and participation in community groups in this same sample of women [18]. We hypothesized that CPT might be effective in addressing felt (i.e. perceived and internalized) stigma among SV survivors due to its focus on cognitive reframing, reducing avoidance of trauma-related cues and social isolation, and specific emphasis on identifying and challenging maladaptive beliefs (such as self-blame and negative beliefs about self and others) that stem from SV experiences [16]. An example of a maladaptive belief is a woman feeling that “I don’t have a voice in my house” on account of being raped. In a prior study of rape survivors in the United States, maladaptive beliefs were found to significantly change as a function of CPT [19]. Further, by helping survivors identify sources of support or to recognize evidence that disconfirms their beliefs, CPT might address women’s overgeneralized perceptions or negative beliefs about how others view them.

Stigma may also affect survivors’ willingness to join or participate fully in group therapies. Stigma related to intimate partner violence and SV in general has been described by women as interfering with daily functioning and care seeking, leading to self-isolation [5, 20]. For example, shame over being raped has been described as a reason for delaying seeking medical care in DRC [21]. In the United States, mental health-related stigma has shown a negative association with ability or willingness to continue pharmacological treatment once initiated [22]. Disruption in care seeking for a variety of problems associated with sexual violence may be a mechanism by which stigma negatively impacts overall health outcomes [23, 24]. We therefore hypothesized that enacted and felt stigma could moderate the effect of CPT on SV survivors’ mental health.

Given the dearth of research on the anti-stigma potential of counseling programs, the multifaceted negative consequences of stigma for SV survivors, and the promise of CPT as an effective intervention, we sought to answer the following research questions: what is the impact of group-CPT on felt stigma among survivors of sexual violence in Eastern DRC; and, does enacted or felt stigma moderate the effect of CPT on mental health?

## Methods

This secondary analysis uses data from a prior randomized controlled trial of lay-worker administered group Cognitive Processing Therapy for female SV survivors in Eastern DRC. Detailed information on trial design is available in a report of primary trial outcomes: posttraumatic stress and combined depression and anxiety [17]. In brief, sixteen villages (14 in South Kivu and 2 in contiguous areas of North Kivu) were purposefully selected out of 23 in which a psychosocial assistant (PSA) was providing supportive counseling to female SV survivors through one of three non-governmental organizations (NGOs) supported by the International Rescue Committee. PSA availability and whether a village could be consistently and safely accessed guided selection. These villages were grouped by location and language and cluster randomized to cognitive processing therapy (CPT; *n* = 7) or a continuation of individual support (IS; *n* = 8). One village was excluded post-randomization due to concerns about its PSA’s ability to provide CPT.

Women were recruited in December 2010 by PSA review of current and former client lists. To be eligible, women needed to (a) have witnessed or experienced SV (“rape” in local translations); (b) be experiencing poor mental health (defined as a score of at least 55 on the total symptom scale, equivalent to an average response of 1 to scale items); and (c) be experiencing some impairment in daily functioning (defined as a score of 10 on the functioning scale, equivalent to indicating at least some dysfunction on half the scale items). Women were excluded if they expressed suicidal ideation that was deemed to need immediate intervention. In areas randomized to CPT, recruitment ceased when 28–30 eligible women were identified in each village. Women closest to the NGO offices were then selected to fill three groups of up to 8 women per village. In IS villages, all eligible women were invited to join the study [17].

Follow-up assessments administered by research assistants masked to treatment assignment took place within one month of the end of CPT and again six months later. Oral informed consent was obtained from all individual participants included in the study. The Johns Hopkins Bloomberg School of Public Health and the Kinshasa School of Public Health Institutional Review Boards provided ethical approval.

### Intervention conditions

Cognitive processing therapy (CPT) is a manualized evidence-based psychotherapy developed specifically for treating mental health problems in rape survivors. To improve the reach of the intervention in DRC where the gap between available services and need is large, CPT was delivered in a group format. In addition, based on findings of similar efficacy [25], a version of CPT without a trauma narrative component was used. Treatment occurred over 12 sessions (of approximately 2 h in length), the first of which was delivered individually. PSAs in villages randomized to CPT received two weeks of training from US-based practitioners. A multi-tiered apprenticeship structure (described elsewhere [26]) was used to support and monitor CPT implementation with oversight by local and US-based supervisors. For the individual support (IS) arm, PSAs continued to be available to women with the same psychosocial support services that they provided before study initiation, including referrals for medical, economic, and legal problems.

### Instruments

The study instrument was translated into five local languages (Kibembe, Kifuliro, Kihavu, Mashi, and Swahili) and included the following sections: demographics, trauma exposure, daily functioning, and psychosocial and mental health problems.

#### Demographics

Participants self reported their age, language, marital status, the total number of people living in their household, and whether they had lived in the same village for 10 years or more. For analysis, marital status was treated as a binary variable of currently married or not.

#### Trauma exposure

Women self-reported whether they had either (a) witnessed or (b) experienced the following events: sexual violence (locally translated as rape), a severe violent attack such as a beating or attack with a weapon, looting or burning of home or property, abandonment or having been thrown out, and abduction. Women also reported whether they had witnessed murder. Two composite variables were created by separately summing the types of traumas experienced (maximum possible score of 5) and witnessed (maximum possible score of 6).

#### Functioning

Following a process described elsewhere [27], a 20-item local functionality inventory was created based on participants’ descriptions of what activities women needed to be able to complete daily to support themselves, their families, and their communities (see Additional file 1 for a full list of activities). Participants indicted how much difficulty they experienced in doing each activity on a Likert scale of 0 (none) to 4 (often cannot do). Item responses were averaged so a woman’s score could range from 0 to 4, with higher scores reflecting greater impairment. Cronbach’s alpha for the functioning scale was 0.93 [17].

#### Psychosocial and mental health problems

Women in three Eastern DRC communities initially described mental health and psychosocial problems experienced by SV survivors during a qualitative study. Participants in this qualitative study constituted a separate sample from the women enrolled in the RCT. Based on these findings, a 55-item mental health symptom and psychosocial problem questionnaire was created that included 15 depression and 10 anxiety items from the Hopkins Symptom Checklist-25 (HSCL), 16 posttraumatic stress items from the Harvard Trauma Questionnaire (HTQ), and additional items described in the qualitative study not covered by these measures (see [17] for a list of all items). The HSCL-25 and HTQ scales were used to measure the primary outcomes of combined depression and anxiety and posttraumatic stress in the original RCT analysis. For the HSCL and HTQ, women were asked to indicate on a Likert scale how often in the last four weeks they experienced each problem ranging from 0 (not at all) to 3 (a lot). Responses to scale items were averaged so mental health symptom scores could range from 0 to 3 with a higher score indicating greater severity. For this secondary analysis, the HTQ item “feeling detached of withdrawn from others” and the HSCL item “feelings of worthlessness, no value” were excluded from the average posttraumatic stress and combined depression and anxiety average scores respectively due to the inclusion of these items on the felt stigma scale (see below). Cronbach’s alpha for the revised HTQ and HSCL scale were 0.87 and 0.89, respectively.

#### Felt and enacted stigma

The stigma scale development and testing process, conducted with baseline data from all women who indicated having directly experienced sexual violence in this sample (*n* = 383), is described in detail elsewhere [28]. Briefly, an initial exploratory factor analysis was conducted using 16 psychosocial problems mentioned by participants that were not captured on the combined depression and anxiety or PTSD scale. Six of these 16 items loaded together on one factor, distinct from items representing more general mental distress such as anger, being cold, or thinking too much. To support the content validity of the measure, three items from HSCL or HTQ (two of which were ultimately retained) and six on traumatic experiences (four of which were ultimately retained) were combined with these 6 initial items and included in further factor analyses. Ultimately, these analyses supported the existence of two related but distinct constructs: one, composed of experiences of rejection and discrimination, we termed enacted stigma (4-items); and a second, composed of indicators of perceived and internalized stigma, we termed *felt stigma* (8-items) (see Additional file 1 for a list of items) [28].

For each felt stigma item, women were asked to indicate on a Likert scale how often in the last four weeks they experienced each problem ranging from 0 (not at all) to 3 (a lot). Item responses were averaged so felt stigma scores could range from 0 to 3 with a higher score indicating greater severity. Cronbach’s alpha for the felt stigma scale was 0.86, indicating strong internal consistency [28]. Data on enacted stigma was only collected at baseline. The enacted stigma scale asked women to indicate whether they had ever experienced particular traumatic events. Participant responses were averaged and rescaled to range from 0 to 10 for ease of interpretability. The Kuder-Richardson coefficient for the enacted stigma scale was 0.68, indicating borderline acceptable internal consistency [28].

### Analysis

Our sample included women who reported witnessing but not directly experiencing SV (*n* = 22, < 6%) and women who reported a SV experience. We chose to include both sets of women because, as women were recruited from the rosters of organizations providing services to survivors of sexual violence, it is likely that women who only indicated witnessing violence had (a) in actuality also experienced SV or (b) still experienced the effects of SV (including stigma) to a certain extent as witnesses. Longitudinal maximum likelihood estimated mixed-effect linear regression models with a robust variance estimator were used for all analyses with an intent-to-treat approach. Random effects included the participant (to account for multiple measures over time), CPT group, and village. Differences in baseline demographics by treatment group were assessed using Student’s *t*-, Wilcoxon rank-sum, and Pearson’s chi-squared tests. Linear regression was used to assess predictors of change in felt stigma over time. Results from these tests and consistency with prior analyses of data from this trial [17, 18] guided covariate selection.

Multiple imputation with chained equations (MICE) [29] was used to account for item-level missing data and loss to follow up. Out of 405 participants, 135 (33%) and 92 (23%) missed assessments at the first and second follow up respectively (participant flow diagram is available; [17]). One item on the enacted stigma sub-scale was only relevant to married women and another only to women with children (see Additional file 1). If a woman’s response was missing for more than two items, her enacted stigma score was considered missing (*n* = 22, 5.43%).

For assessing the impact of CPT on felt stigma, time and treatment group were included as fixed effects. Treatment effect was the difference in change in felt stigma score over time by treatment group (time by treatment group interaction). For moderation analyses, we examined the difference between intervention and control participants’ mean changes on study outcome scales (HSCL, HTQ, and functioning) over time by level of enacted and felt stigma reported at baseline (three-way interaction between intervention arm, time, and stigma variable). Cohen’s d effect sizes were calculated by standardizing regression coefficients by the pooled standard deviation of the outcome at baseline [30]. All analyses were implemented with Stata 13 [31].

## Results

### Demographics and baseline characteristics of study women

Women randomized to group-CPT were older on average (37 vs. 34 years of age), more likely to speak Mashi or Kihavu, and a greater proportion were currently married (59% vs. 43%) (Table 1). Women randomized to CPT were also, on average, living in larger households at baseline (7.4 vs. 6.8 people) and more likely to have lived in their village for ten years or more (75% vs. 60%). Overall, women had approximately 2 years of formal education and were responsible for an average of four children. Women assigned to continue IS reported greater functional impairment, combined depression and anxiety, and posttraumatic stress symptoms than women assigned to CPT at baseline; however, they reported experiencing and witnessing fewer types of traumas than women in the CPT arm (3.4 vs. 3.9 and 4.1 vs. 5.2, respectively). Across the trial arms, 40 women (10%) reported knowing the person(s) who perpetrated the act of sexual violence. There was no significant difference in enacted stigma by group, though women in IS did report a slightly greater number of discrimination experiences. As reported previously, 141 women (90%) in CPT completed treatment (i.e. attended at least 9 sessions). In the IS arm, 182 women (73%) met with the APS at least once; on average, these 182 women attended 5 sessions with the APS [17].

**Table 1:** Demographics, traumatic experiences, and mental health of women in Democratic Republic of Congo at study baseline, April 2011

Variable	CPT (*n* = 157)	IS (*n* = 248)
Demographic characteristics		
Age in years, Mean (SD)*	36.89 (13.44)	33.77 (12.43)
Years of education completed, Mean (SD)	1.76 (2.76)	2.25 (3.14)
Number of people living in home, Mean (SD)*	7.41 (3.15)	6.81 (3.32)
Number of children responsible for, Mean (SD)	3.96 (2.67)	4.06 (2.76)
Marital Status, No. (%)*		
Single	20 (12.74)	35 (14.11)
Married	93 (59.24)	107 (43.15)
Divorced	1 (0.65)	11 (4.44)
Separated	19 (12.10)	43 (17.34)
Widowed	24 (15.29)	52 (20.97)
Living in territory of origin, No. (%)	130 (82.80)	194 (78.23)
Lived at current home for 10 years or more, No. (%)*	118 (75.16)	148 (59.68)
Language, No. (%)*		
Kibembe	0 (0.0)	46 (18.55)
Kifuliro	30 (19.11)	64 (25.81)
Kihavu	58 (36.94)	81 (32.66)
Mashi	45 (28.66)	0 (0.0)
Swahili	24 (15.29)	57 (22.98)
Perpetrator of sexual violence known to woman^a^	12 (8.11)	28 (11.91)
Trauma and Discrimination Experiences		
Average different traumas experienced, Mean (SD)*	3.91 (1.08)	3.36 (1.36)
Average different traumas witnessed, Mean (SD)*	5.20 (1.28)	4.06 (1.96)
Enacted stigma, Mean (SD)	4.67 (3.43)	5.13 (3.50)
Mental health		
Functioning score, Mean (SD)*	1.65 (0.69)	2.48 (0.82)
Depression and anxiety score, Mean (SD)*	1.95 (0.51)	2.18 (0.46)
Posttraumatic stress score, Mean (SD)*	1.86 (0.58)	2.21 (0.49)

Note: *CPT* Cognitive Processing Therapy, *IS* individual support, *SD* standard deviation; *Between arm difference significant at *p* < 0.05. ^a^Denominator is comprised of the 383 women who indicated personally experiencing sexual violence

### The effect of cognitive processing therapy (CPT) on felt stigma

Overall, women at baseline had a mean felt stigma score of 1.98, equivalent to experiencing a moderate amount of felt stigma on average. Women randomized to IS reported more severe felt stigma than women in CPT, a difference that reached statistical significance (Table 2). While women in both arms experienced a reduction in felt stigma over time, participants in group-CPT experienced a significantly greater decline in felt stigma relative to IS participants at the end of treatment (B = − 0.44, *p* value = 0.02). This magnitude but not the statistical significance of this effect was maintained six months post-treatment (B = − 0.45, *p* value = 0.12).

**Table 2:** Effect of CPT on felt stigma among women in Democratic Republic of Congo at end of treatment and after six-month maintenance period, April 201 l–February 2012 (*n* = 405)

Time point	CPT^a^ Mean (SD)	IS^a^ Mean (SD)	b (SE)^b^	B^c^	*P*-value
Baseline	1.72 (0.67)	2.15 (0.62)			< 0.001^d^
Post intervention	0.57 (0.68)	1.60 (0.82)	−0.30 (0.13)	− 0.44	0.024
6 mo. post-intervention	0.60 (0.65)	1.43 (0.74)	−0.31 (0.20)	−0.45	0.119

Note: *CPT* Cognitive Processing Therapy, *IS* individual support, *SD* standard deviation, *SE* standard error. ^a^Unadjusted mean scores. ^b^β is the coefficient for the interaction between treatment group (CPT or IS) and assessment time point and represents the difference in the change in felt stigma over time between women in the CPT and IS arms. Estimated using longitudinal mixed-effect linear regression; random effects included participant, CPT group, and village. Model covariates were baseline age, marital status (currently married yes or no), language, having lived in the current village for at least 10 years or less, total number of people living in the household, number of types of traumas experienced and witnessed, average baseline functioning score, and average baseline score on all mental health symptom items not included on the felt stigma scale. ^c^Cohen’s D effect size standardized using the pooled baseline standard deviation of the felt stigma outcome. ^d^*P*-value is for the Wilcoxon rank-sum test of difference in felt stigma mean score at baseline by treatment group

### Moderation of CPT effectiveness for mental health problems by stigma

Reported felt stigma at baseline did not significantly moderate the effect of CPT on combined depression and anxiety (b = − 0.07; 95% confidence interval (CI): − 0.40, 0.27) at the end of treatment (see Table 3). Higher felt stigma at baseline was associated with a greater, but non-significant, treatment effect of CPT relative to IS for posttraumatic stress (b = − 0.18; 95% CI: -0.49, 0.14) and functional impairment (b = − 0.17; 95% CI: -0.57, 0.24) at the end of treatment. Enacted stigma at baseline did not significantly moderate the effect of CPT on functional impairment (b = − 0.02; 95% CI: -0.07, 0.04) or posttraumatic stress (b = 0.02; 95% CI: -0.04, 0.07). Higher reported experiences of enacted stigma at baseline were most strongly associated with a reduction in the effect of CPT relative to IS for combined depression and anxiety (b = 0.04; 95% CI: 0.005, 0.08), though this difference was also non-significant.

**Table 3:** Moderation of the effect of CPT on the mental health of women in Democratic Republic of Congo by stigma reported at treatment initiation, April 2011–February 2012 (*n* = 405)

Outcome	Felt stigma^e^ b (SE) ^a^	Felt stigma *p*-value	Enacted stigma^e^ (SE) ^a,b,c^	Enacted stigma p-value
Depression and Anxiety ^d^				
Post intervention	−0.07 (0.17)	0.71	0.04 (0.02)	0.08
6 mo. post-intervention	0.01 (0.18)	0.94	0.03 (0.03)	0.36
Posttraumatic stress ^d^				
Post intervention	−0.18 (0.16)	0.27	0.02 (0.03)	0.56
6 mo. post-intervention	0.01 (0.20)	0.95	−0.004 (0.03)	0.88
Functioning^e^				
Post intervention	−0.17 (0.21)	0.42	−0.02 (0.03)	0.55
6 mo. post-intervention	0.18 (0.25)	0.48	−0.01 (0.04)	0.73

Note: *CPT* Cognitive Processing Therapy, *SE* standard error. ^a^b is the beta-coefficient for the three-way interaction between treatment group (CPT or IS), assessment time point and moderating variable (felt-stigma or enacted stigma scale score) and represents the difference associated with a one unit increase in stigma score of the change in outcome over the time period between women in the CPT and IS arms. Estimated using longitudinal mixed-effect linear regression; random effects included participant, CPT group, and village. Model covariates were baseline age, marital status (currently married yes or no), currently pregnant, language, having lived in the current village for at least 10 years or less, total number of people living in the household, number of children for which the participant is responsible, and number of types of traumas experienced and witnessed. ^b^Sample size for the analysis of enacted stigma as a moderator is reduced to *n* = 383 due to some women missing more than 50% of the scale as one item was only relevant to married women (“rejected by husband”) and another to those with children (“forced to live away from your children”). ^c^For estimation of beta coefficients for the outcome of posttraumatic stress, 10 imputations were used instead of 11 due to a convergence failure in one imputed data set. ^d^The HTQ item “feeling detached of withdrawn from others” and the HSCL item “feelings of worthlessness, no value” were excluded from the average posttraumatic stress and combined depression and anxiety average scores respectively due to the inclusion of these items on the felt stigma scale. ^e^Locally developed scale, based primarily on qualitative research with women in DRC

## Discussion

We sought to explore the potential of a group-delivered evidence-based psychotherapy, Cognitive Processing Therapy, to reduce felt (i.e. internalized and perceived) stigma among female survivors of sexual violence in eastern DRC. Experiences and feelings of stigma were common among survivors at baseline. Group CPT participants experienced moderately greater reductions in felt stigma relative to control participants who only had access to basic individual psychosocial support. The magnitude, though not the statistical significance, of this effect was maintained six months-post treatment. Neither felt nor enacted stigma significantly moderated the effect of CPT on mental health outcomes or functionality. However, women reporting more discrimination at baseline did experience slightly lower treatment effects.

The group format of the intervention is one possible mechanism behind the reduction observed in felt stigma; findings on effects of group psychosocial interventions on stigma are mixed. Some limited evidence exists that group-delivered Acceptance and Commitment Therapy, a psychotherapy based in principles of mindfulness, can reduce substance abuse-related internalized stigma [32]. Conversely, a study of group Cognitive Behavioral Therapy found improvements in self-esteem among people with schizophrenia, but not perceived stigma [33]. The mixed nature of these findings from the broader literature suggests that while social contact may be an important factor to consider in efforts to reduce stigma, the nature of that contact, the characteristics of the participants, and the content of group activities must be given critical consideration.

An emphasis on cognitive restructuring, a key component in CPT, is another potential mechanism of the observed impact on felt stigma. This focus may explain differences in stigma reduction effects observed between CPT and other trauma-focused treatments that place greater emphasis on gradual desensitization or exposure [25, 34]. CPT was found to be more effective than Prolonged Exposure Therapy in reducing guilt related to hindsight bias (feeling like the person could have done something to stop what happened) and lack of justification (for what they did surrounding the trauma) among SV survivors in the US [25]. A second exposure based therapy for PTSD, Narrative Exposure Therapy, delivered individually to child soldiers in northern Uganda was found to have no impact on perceived stigma [34]. This supports the idea that cognitive restructuring may be a skill that helps survivors’ not only deal with their own and others’ beliefs regarding trauma experiences and mental disorders, but other interrelated domains important to overall wellness.

A group-based microfinance program (with no psychotherapeutic components) in the same area of DRC also resulted in a reduction in felt stigma for female survivors of sexual violence, but this reduction was smaller in magnitude [35]. Although one potential pathway of this effect could have been social interaction and acceptance by women in the group, other pathways specific to economic activity (e.g. empowerment) could also explain this change. Differences in size of the effect between CPT and this microfinance program could be due to the programs impacting different aspects of the stigma experience. For instance, a microfinance program might be more effective in reducing stigma specifically associated with perceptions of exclusion from economic activities or non-specific feelings of low worth. For individuals who primarily experience stigma as shame or blame, this type of program may not be effective. Alternatively, as a general skill with potential broad utility, cognitive restructuring may be effective in helping women address multiple components of the stigma experience.

The lower degree of felt stigma reported by CPT participants was largely maintained six months post-treatment, though the effect lost statistical significance. One potential area for future research is whether booster sessions may be needed to promote the long-term well-being of women in a context of continued adversity. As women may continue to be exposed to acts of SV and enacted stigma, the feasibility of combining CPT with a broader community-based campaign for social change, and whether this type of multilevel intervention could bolster the moderately sized reduction in stigma observed in this study, is worthy of investigation. As the enacted stigma scale items asked about having ever experienced these acts of discrimination, we were not able to assess the effect of CPT on enacted stigma since we could not accurately detect change in this outcome. Future study should assess if CPT has the potential to impact other forms of stigma.

Our lack of significant findings regarding moderation of CPT treatment effects by enacted or felt stigma may be due to several different factors. Our primary hypothesis was that both forms of stigma might impact care seeking, as in DRC women who experienced stigma on average took longer to seek services for sexual violence than those who did not [21]. Given that our sample was drawn from women who had already chosen to seek support services specifically designed for sexual violence survivors, these women may have been less likely to delay or decide not to seek care when invited by trusted providers a second time, inhibiting our ability to see an effect. We also hypothesized that ultimately stigma might impact treatment success because it could lead to lower engagement in care. This hypothesis was based on a prior study in the US that found lower perceived stigma predicted greater adherence to antidepressant medication [22]. Overall, women in CPT were highly engaged in therapy. Our hypothesis therefore could simply be incorrect in this context where the need is great and opportunities for mental health services are otherwise practically non-existent.

### Limitations

As this represents a secondary analysis of an evaluation of the effect of CPT on mental distress, the original trial was not designed to assess stigma as an outcome. Accordingly, there may be important parts of the stigma experience in this context that are not included in the measure used. As an example, survivors in DRC have described rejection as a complex concept that extends beyond physical separation from family and partners to include emotional and financial changes in relationships [5]. As most of the items on our stigma scales arose from qualitative research in DRC, our measure may also be specific to the context. Further, as the enacted stigma scale included some items only relevant to women who have children or are married, findings regarding moderation of mental health treatment effects of CPT by enacted stigma may have limited generalizability. Women were recruited for this trial through services providing psychosocial assistance to sexual violence survivors, and thus our results may not be generalizable to non-careseeking women.

As felt stigma includes an individual’s own assessment of their worth and how others view them, it is likely that factors related to mental health problems (e.g. negative affect or attribution bias) can shape felt stigma. This makes it challenging to distinguish between general emotional distress and internalized or perceived stigma in the measurement of the construct. We did find in earlier sensitivity analyses that depression and felt stigma items largely loaded onto distinct latent constructs in an exploratory factor analysis [28]. In addition, after removing the three items from the felt stigma scale that we hypothesized would most strongly overlap with aspects of depression (“feelings of worthlessness”, “wanting to avoid others or hide”, and “feeling detached from others”), our findings were not meaningfully changed (results available upon request). It is also notable that the effect of CPT on felt stigma was attenuated relative to the effects on depression, anxiety, and trauma, which we expected given the intervention was designed to explicitly address mental health problems rather than stigma specifically. A further limitation is our inability to assess the long-term impact of the effects of CPT on felt stigma.

## Conclusions

Despite these limitations, our findings suggest that psychotherapies may be an effective tool for reducing feelings of stigma in women affected by sexual violence. Important for practitioners, group mental health therapies delivered to sexual violence survivors may be effective regardless of the level of stigma women perceive or internalize or past experiences of discrimination. Future research should compare individual and group delivered therapies to tease out the impact of group participation from that of the actual psychotherapy elements (e.g., cognitive restructuring). In addition to exploring the mechanisms by which CPT may impact stigma, another important direction for future research is assessing whether reductions in stigma may facilitate long-term improvements in women’s mental health.

## Additional file

Additional file 1: List of items included in locally-developed scales. (DOCX 68 kb)

## Abbreviations

CPT: Cognitive Processing Therapy
DRC: Democratic Republic of Congo
IS: Individual support
NGO: Non-governmental organization
PSA: Psychosocial assistant
PTSD: Posttraumatic stress disorder
SV: Sexual violence
