# Supplementary material for: The impact of Cognitive Processing Therapy on stigma among survivors of sexual violence in eastern Democratic Republic of Congo: results from a cluster randomized controlled trial
Source: Confl Health. 2018 Feb 12;12:1. doi: 10.1186/s13031-018-0142-4 (PMC5808396; doi:10.1186/s13031-018-0142-4)
Supplement: Supplementary file 1 — List of items included in locally-developed scales. (DOCX 68 kb) [file 13031_2018_142_MOESM1_ESM.docx]

**The impact of Cognitive Processing Therapy on stigma among survivors of sexual violence in Eastern Democratic Republic of Congo: Results from a cluster randomized controlled trial**

Sarah M Murray^1^, Jura Augustinavicius^1^, Debra Kaysen^2,3^, Deepa Rao^2,3^, Laura Murray^1^, Karin Wachter^4^, Jeannie Annan^5^, Kathryn Falb^5^, Paul Bolton^1,6^, Judith Bass^1^

**Corresponding author:** Sarah M Murray, Johns Hopkins Bloomberg School of Public Health**,** Department of Mental Health**;** [smurray9@jhu.edu](mailto:smurray9@jhu.edu)

**List of items used to measure stigma**

| *Felt stigma* | *Enacted stigma* |
| --- | --- |
| Feelings of worthlessness | Abandoned/thrown out |
| Feeling detached from others | Rejected by family |
| Feeling badly treated by family | Rejected by husband |
| Feeling badly treated by community | Forced to live away from your children |
| Feeling shame |  |
| Feeling rejected by everybody |  |
| Feeling stigma |  |
| Wanting to avoid others or hide |  |

**List of activities used to measure functional impairment**

| Cultivating/farming |
| --- |
| Trading or other ways of making money |
| Cooking |
| Looking after children |
| Giving advice to family members |
| Giving advice to other community members |
| Exchanging ideas with others  Raising/breeding animals  Other types of manual labor |
| Uniting with other community members to do tasks for the community |
| Uniting with other family members to do tasks for the family |
| Socializing with others in the community |
| Asking/getting help from people or an organization when you need it |
| Making important decisions about daily life |
| Taking part in family activities or events |
| Taking part in community activities or events |
| Learning new skills or knowledge  Concentrating on your tasks/responsibilities  Interacting or dealing with people you do not know  Attending church or mosque as usual |
